# Sick Rooms

**Published:** 1873-08

**Authors:** 


### SICK ROOMS

should face the south, if possible, so as to secure the sunlight for a greater part of every day, keeping the atmosphere healthful, light, pure and dry.

A good ventilation is absolutely necessary at all seasons, and this ventilation should be kept up day and night, and at night time more particularly, because the air is heavy and damp, hence contains less nutriment ; in the day time the doors are constantly opening, and if the fireplace is kept free there is a good draught up the chimney, especially if there is a fire in the room.

In warm weather a steady draught may be always kept in operation by placing a burning lamp or candle in the fireplace, close to the back part.

There is an almost universal impression that air, to be pure, must be cold ; this is not so ; the sick need pure air, but if it comes upon them cold, ill results are sure to follow. Hence, in cold weather, the air coming from without should be at the farthest point from the invalid, so that it will be more or less warmed before reaching him. In very cold weather the window may be raised a little from the bottom—half an inch only.

If a room is heated by a stove or register, the fireplace should be kept open all the time ; if there be no fireplace, then an opening should be made into the flue of the house, or the transom over the door should be kept open, and a sash hoisted or lowered as nearly opposite as possible.

